# Integrated toxicogenomic and single-nucleus transcriptomic analyses prioritize SDHB and NDUFAB1 in mitochondrial respiration-associated remodeling during cisplatin-induced acute kidney injury

**DOI:** 10.3389/fcell.2026.1939354

**Published:** 2026-10-01

**Authors:** Dianjie Zeng, Yunhui He, Huijie Hu, Xuzheng Wang, Changkun Huang

**Affiliations:** 1 Department of Urology, The Second Xiangya Hospital at Central South University, Changsha, Hunan, China; 2 Capital Medical University, Beijing, China; 3 Department of Nephrology, The Third Xiangya Hospital at Central South University, Changsha, Hunan, China; 4 Guangdong Medical University, Dongguan, Guangdong, China

**Keywords:** cisplatin-induced acute kidney injury, mitochondrial remodeling, NDUFAB1, proximal tubular epithelial cells, SDHB, single-nucleus RNA sequencing

## Abstract

**Background:**

Cisplatin-induced acute kidney injury (Cis-AKI) is a multifactorial nephrotoxic syndrome involving proximal tubular injury, oxidative and inflammatory stress, mitochondrial dysfunction, metabolic disturbance, and regulated cell death. However, the molecular targets linking cisplatin-associated toxicogenomic changes to AKI remain incompletely characterized.

**Methods:**

We integrated network toxicology, transcriptomic analysis, single-nucleus RNA sequencing, and *in vitro* experiments to investigate candidate molecular targets in Cis-AKI. Cisplatin-associated toxicogenomic genes were intersected with AKI-related genes, and the shared candidates were analyzed using functional enrichment, protein–protein interaction networks, CytoHubba, and MCODE. Prioritized genes were further examined in a cisplatin-induced mouse Acute kidney injury transcriptomic dataset, a human AKI kidney single-nucleus dataset, proximal tubular pseudotime analysis, and cisplatin-treated BUMPT proximal tubular cells. NicheNet analysis, knockout phenotype annotation, miRNA prediction, candidate compound screening, and molecular docking were also performed.

**Results:**

A total of 214 shared genes were identified between the cisplatin-associated and AKI-related gene sets. These genes were mainly enriched in cellular respiration, electron transport, oxidoreductase activity, respiratory-chain complexes, and oxidative phosphorylation. Network analysis prioritized SDHB and NDUFAB1 within the respiration-associated gene network. In the cisplatin-induced mouse AKI dataset, Aco2, Aco1, Sdhb, and Aldh2 were significantly upregulated under stringent criteria, whereas additional respiration- and metabolism-related genes, including Ndufab1, met a secondary differential expression threshold. Human single-nucleus analysis revealed distinct cell-type-specific expression patterns of SDHB and NDUFAB1, with increased SDHB and decreased NDUFAB1 expression in proximal tubular cells. Pseudotime analysis showed a more pronounced late-stage increase in SDHB, whereas NDUFAB1 displayed weaker dynamics. In cisplatin-treated BUMPT cells, Havcr1 and Lcn2 were increased, Bcl2 was decreased, and Casp3 was increased. Among the selected candidate genes, Sdhb, Aco2, Aldh2, Ndufs2, and Uqcrc1 were upregulated, whereas Ndufab1 showed a modest decrease. NicheNet analysis further suggested that TGFB1-related signaling may contribute to proximal tubular transcriptional remodeling.

**Conclusion:**

This study prioritizes SDHB and NDUFAB1 within a respiration-associated gene network in Cis-AKI and demonstrates that they exhibit distinct expression patterns during proximal tubular injury. These findings highlight respiration-associated remodeling and proximal tubular state transition in cisplatin-induced kidney injury.

## 1 Introduction

Acute kidney injury (AKI) is a clinically common syndrome, particularly among hospitalized and critically ill patients, characterized by an abrupt decline in renal excretory function and disruption of fluid, electrolyte, and metabolic homeostasis (Kellum et al., 2021; Zarbock et al., 2024; Pérez-Aizpurua et al., 2024). According to the Kidney Disease: Improving Global Outcomes (KDIGO) framework, AKI is diagnosed by an acute increase in serum creatinine or a reduction in urine output within a defined short-term window (Ostermann et al., 2020). AKI is associated not only with short-term mortality and prolonged hospitalization but also with incomplete recovery and maladaptive repair, which may promote AKI-to-CKD transition and increase the long-term risk of chronic kidney disease, cardiovascular complications, and end-stage kidney disease (See et al., 2019; Li et al., 2024; Jiang et al., 2024). Despite its clinical burden, current management remains largely supportive, including hemodynamic optimization, avoidance of nephrotoxins, fluid and electrolyte management, and kidney replacement therapy in severe cases (Wald et al., 2022). Mechanism-oriented therapeutic strategies remain limited (Ronco et al., 2019).

Cisplatin is widely used for the treatment of solid tumors, including lung, head and neck, ovarian, bladder, and testicular cancers. However, nephrotoxicity remains a major dose-limiting adverse effect (Holditch et al., 2019). Cisplatin preferentially accumulates in renal tubular epithelial cells, particularly proximal tubular cells, through organic cation and copper transport systems (Ciarimboli et al., 2005; Filipski et al., 2009). Once internalized, cisplatin can induce nuclear and mitochondrial DNA damage, respiratory-chain impairment, ATP depletion, excessive reactive oxygen species production, endoplasmic reticulum stress, inflammatory signaling, apoptosis, necroptosis, and other forms of regulated cell death (Airik et al., 2024; Su et al., 2024; Pei et al., 2022; Song et al., 2026a). These overlapping processes indicate that cisplatin-induced AKI (Cis-AKI) is a multi-target and multi-pathway injury state rather than a consequence of a single molecular lesion.

Although mitochondrial dysfunction is a well-established feature of cisplatin nephrotoxicity, the specific molecular components linking cisplatin-related toxicogenomic perturbations to AKI remain incompletely characterized. In particular, it remains unclear which respiration- and metabolism-related genes show reproducible injury-associated changes across complementary biological contexts and whether these changes can be localized to specific renal cell populations and proximal tubular injury states. Conventional transcriptomic studies can identify disease-associated expression changes, but their results may be influenced by dataset-specific context, sampling time, tissue composition, and analytical thresholds. In contrast, network toxicology provides a disease-anchored and exposure-specific strategy for linking chemicals, genes, biological pathways, and disease phenotypes (Sutherland et al., 2018; Liu et al., 2025a; Song et al., 2026b; Li Z. et al., 2026; Zhang et al., 2026). By integrating toxicogenomic evidence with disease-gene knowledge, this approach can prioritize candidate molecular links between a nephrotoxic exposure and its pathological outcome.

Database-based target prioritization alone, however, cannot define the cellular distribution or injury-associated expression patterns of candidate genes. Cis-AKI involves multiple renal compartments, although proximal tubular cells represent a major site of cisplatin accumulation and injury (Liu et al., 2025b). Evaluation across complementary datasets and experimental models may therefore help determine whether prioritized genes are associated with cisplatin-induced transcriptional changes, specific renal cell populations, and proximal tubular injury states (Hukriede et al., 2022; Zhou et al., 2026; Zhu et al., 2025). Integrating network analysis with cisplatin-induced mouse transcriptomics, human AKI single-nucleus profiling, proximal tubular trajectory analysis, and cisplatin-treated tubular cell experiments may provide a more complete view of respiration- and metabolism-related remodeling associated with Cis-AKI.

In this study, we integrated network toxicology, transcriptomic analysis, human AKI kidney single-nucleus profiling, and *in vitro* qRT-PCR analysis to investigate candidate molecular targets in Cis-AKI. Cisplatin-related toxicogenomic genes were intersected with AKI-associated genes, followed by functional enrichment, protein–protein interaction network analysis, CytoHubba ranking, and Molecular Complex Detection module analysis. Prioritized genes were then examined using a cisplatin-induced mouse AKI transcriptomic dataset, human AKI single-nucleus kidney data, proximal tubular pseudotime analysis, and qRT-PCR in cisplatin-treated BUMPT cells. Additional analyses included NicheNet, mouse knockout phenotype annotation, miRNA prediction, candidate compound screening, and molecular docking to further characterize the prioritized targets (Figure 1).

**FIGURE 1:**
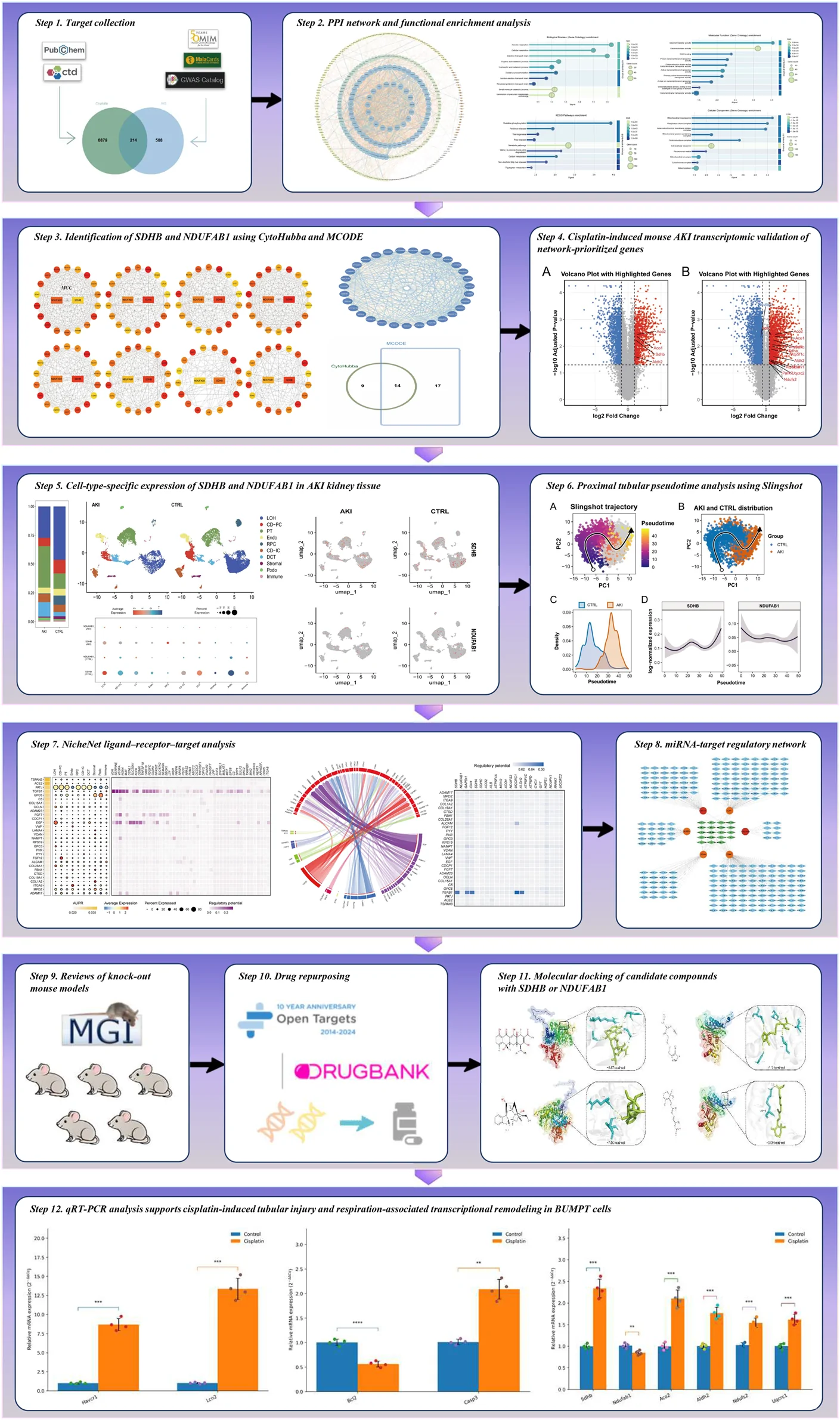
Flowchart of this study.

## 2 Methods

### 2.1 Acquisition of cisplatin-associated toxicogenomic genes and AKI-related genes

The chemical structure of cisplatin was retrieved from PubChem (https://pubchem.ncbi.nlm.nih.gov/), and cisplatin-associated chemical–gene interaction records were obtained from the Comparative Toxicogenomics Database (CTD; https://ctdbase.org/). The records were restricted to *Homo sapiens*, gene symbols were harmonized to official human gene symbols, and duplicate entries were removed. Acute kidney injury (AKI)-related genes were collected from OMIM (https://www.omim.org/), MalaCards (https://www.malacards.org/), and the GWAS Catalog (https://www.ebi.ac.uk/gwas/). The union of genes obtained from these three resources was used as the AKI-related gene set for subsequent analyses (Supplementary Table S1).

### 2.2 Identification of candidate Cis-AKI targets

Candidate targets for cisplatin-induced acute kidney injury (Cis-AKI) were defined as genes shared between the cisplatin-associated toxicogenomic gene set and the AKI-related gene set. The overlapping genes were visualized using a Venn diagram and retained for subsequent network and enrichment analyses.

### 2.3 PPI network construction and functional enrichment analysis

The candidate targets were imported into the STRING database (https://string-db.org/), with *Homo sapiens* selected as the organism and a minimum interaction confidence score of 0.4. The resulting protein-protein interaction (PPI) network was visualized in Cytoscape (version 3.10.2) (Shannon et al., 2003). Gene Ontology (GO) and Kyoto Encyclopedia of Genes and Genomes (KEGG) enrichment analyses were performed in R (version 4.3.2) using the clusterProfiler package. Enrichment P values were adjusted using the Benjamini-Hochberg method, and terms with adjusted P < 0.05 were considered significant.

### 2.4 Hub gene prioritization using CytoHubba and MCODE

Hub genes were prioritized using the Cytoscape plugins CytoHubba and Molecular Complex Detection (MCODE). CytoHubba was used to rank nodes in the PPI network using eight topological algorithms, including maximal clique centrality (MCC), maximum neighborhood component (MNC), Degree, edge percolated component (EPC), Closeness, Radiality, Betweenness, and Stress. The top 10 ranked genes from each algorithm were retained, and the number of algorithms supporting each gene was calculated (Zeng et al., 2026a). MCODE was subsequently used to identify densely connected modules within the PPI network. Genes consistently ranked among the top candidates across the eight CytoHubba algorithms and included in the highest-scoring MCODE module were selected as hub genes.

### 2.5 Cisplatin-induced mouse AKI transcriptomic analysis

An independent cisplatin-induced mouse AKI transcriptomic dataset reported by Deng et al. was used as an etiologically matched validation layer (Deng et al., 2026). The dataset included four cisplatin-treated kidney samples and three control kidney samples and was not used for initial target discovery. After normalization and gene annotation, differential expression analysis between the cisplatin-treated and control groups was performed in R (version 4.3.2) using the limma package (Ritchie et al., 2015). Genes with FDR <0.05 and log2FC > 1 were defined as significantly upregulated. A secondary threshold of FDR <0.05 and |log2FC| > 0.5 was used to examine genes with more moderate expression changes (Zeng et al., 2026b). Mouse gene symbols were retained in transcriptomic figures, whereas corresponding human orthologues were used where cross-species comparisons were required. Volcano plots were used to visualize the differential expression results and selected network-prioritized genes.

### 2.6 Mouse knockout phenotype evidence for prioritized targets

Mouse knockout phenotype evidence was obtained from the Mouse Genome Informatics database (MGI; https://www.informatics.jax.org/) using the official MGI_PhenotypicAllele.rpt and ALL_Phenotype.rpt files. For each prioritized gene, one representative knockout model was selected, prioritizing targeted null/knockout alleles and then endonuclease-mediated null/knockout alleles when targeted models were unavailable. Only alleles with specific MGI accession identifiers were retained. Phenotypes were summarized by major affected systems, with emphasis on renal/urinary, metabolic, developmental, survival, and mitochondrial or cellular respiration-related phenotypes. The knockout evidence was interpreted as genetic phenotype context rather than direct proof of Cis-AKI causality.

### 2.7 Single-nucleus transcriptomic analysis and cell-type-specific expression

Human kidney single-nucleus transcriptomic data from AKI and normal kidney tissues reported by Legouis et al. were analyzed to examine the cell-type-specific expression of the prioritized genes (Legouis et al., 2024). Quality control, normalization, highly variable gene identification, dimensionality reduction, clustering, and visualization were performed using Seurat (Butler et al., 2018). Batch effects were corrected using Harmony (Korsunsky et al., 2019). Cell types were annotated using established renal marker genes, published annotations, and the CellMarker 2.0 database (http://bio-bigdata.hrbmu.edu.cn/CellMarker/). The annotated populations included immune cells, endothelial cells (Endo), podocytes (Podo), proximal tubular cells (PT), collecting duct principal cells (CD-PC), loop of Henle cells (LOH), renal progenitor cells (RPC), distal convoluted tubular cells (DCT), collecting duct intercalated cells (CD-IC), and stromal cells. Gene expression was visualized using UMAP feature plots and dot plots showing average expression and the percentage of expressing nuclei.

### 2.8 Proximal tubular pseudotime analysis using Slingshot

Because proximal tubular cells represent a major site of injury in AKI, proximal tubular nuclei were extracted for trajectory analysis. Dimensionality reduction coordinates and cluster labels were used as input for Slingshot to infer lineage structure and assign pseudotime values (Street et al., 2018). The reconstructed trajectory was visualized on reduced-dimensional embeddings, and the distributions of AKI and control cells along pseudotime were compared using density plots. Smoothed expression trends of SDHB and NDUFAB1 along pseudotime were fitted to evaluate their dynamic changes during proximal tubular state transition.

### 2.9 NicheNet ligand-receptor-target analysis

To explore potential upstream intercellular signals regulating proximal tubular transcriptional remodeling, ligand-receptor-target analysis was performed using NicheNet (Browaeys et al., 2020). Proximal tubular cells were defined as receiver cells, and all annotated renal cell types were considered potential sender cells. Genes expressed in more than 10% of cells were identified separately in receiver and sender populations. Candidate ligands were restricted to ligands expressed in sender cells whose corresponding receptors were expressed in proximal tubular cells.

Differentially upregulated genes in AKI proximal tubular cells compared with control proximal tubular cells were used as the gene set of interest. Genes expressed in proximal tubular cells and present in the NicheNet ligand-target prior matrix were used as background genes. Candidate ligand activities were ranked by corrected area under the precision-recall curve (corrected AUPR). Top-ranked ligands were further linked to predicted proximal tubular receptors and target genes. Ligand activity, ligand expression across sender cell types, ligand–receptor interactions, and ligand–target regulatory potential were visualized using heatmaps, dot plots, and circos plots.

### 2.10 miRNA-target regulatory network

Potential upstream microRNAs (miRNAs) targeting SDHB, NDUFAB1, and selected network-prioritized targets were predicted using miRDB (https://mirdb.org/). Predicted miRNA-target pairs were retained based on database-provided prediction scores when applicable and were imported into Cytoscape (version 3.10.2) to construct the miRNA-target regulatory network. Network nodes were annotated as hub genes, additional prioritized targets, single-target miRNAs, or multi-target miRNAs according to their predicted regulatory relationships (Supplementary Table S2).

### 2.11 Candidate compound screening and molecular docking

Candidate compounds annotated in DrugBank (https://go.drugbank.com/) as being associated with SDHB or NDUFAB1 were retrieved for subsequent analysis. Drug name, maximum development phase, action type, associated indications, and DrugBank identifiers were extracted when available. Protein structures of SDHB and NDUFAB1 were obtained from the RCSB Protein Data Bank (RCSB PDB; https://www.rcsb.org/) or predicted structures when experimentally resolved structures were unavailable. Ligand structures were downloaded from PubChem (https://pubchem.ncbi.nlm.nih.gov/) and prepared using a standard molecular docking workflow. Molecular docking was performed using AutoDock Vina (version 1.2.0) (Eberhardt et al., 2021), and binding affinities were reported in kcal/mol. The lowest-energy conformations were visualized using PyMOL (version 2.2.0) (Seeliger and de Groot, 2010). Docking was used as an exploratory assessment of structural compatibility and was not interpreted as evidence of therapeutic efficacy in Cis-AKI.

### 2.12 Cell culture and cisplatin treatment

Mouse BUMPT proximal tubular epithelial cells were cultured in complete growth medium supplemented with 10% fetal bovine serum and 1% penicillin–streptomycin. Cells were maintained in a humidified incubator at 37 °C with 5% CO_2_. For *in vitro* expression validation, cells were treated with cisplatin (Sigma-Aldrich, #P4394) at a final concentration of 20 μM for 16 h. Control cells were cultured under the same conditions without cisplatin treatment. Four independent biological replicates were included in each group.

### 2.13 qRT-PCR analysis

Total RNA was extracted from BUMPT cells using TRIzol Reagent (Invitrogen, #15-596-026) according to the manufacturer’s instructions. Genomic DNA was removed, and cDNA was synthesized using the PrimeScript RT Reagent Kit (TaKaRa, #RR037A). Quantitative PCR was performed using equal amounts of cDNA and SYBR Green Master Mix (Vazyme, #Q111-02). Relative mRNA expression was calculated using the 2^−ΔΔCq method, with Actb used as the internal reference gene. The analyzed genes included six selected candidate genes (Sdhb, Ndufab1, Aco2, Aldh2, Ndufs2, and Uqcrc1), tubular injury markers (Havcr1/Kim-1 and Lcn2/Ngal), and apoptosis-related genes (Bcl2 and Casp3). Primer sequences are provided in Supplementary Table S3, and source data are provided in Supplementary Table S4.

### 2.14 Statistical analysis for *in vitro* experiments

Four independent cell-culture experiments were performed for each group (n = 4 biological replicates). Data are presented as mean ± standard deviation (SD). Statistical comparisons of qRT-PCR results were performed using ΔCq values, whereas relative expression levels are presented as 2^−ΔΔCq fold changes. Normality and homogeneity of variance were assessed using the Shapiro–Wilk and Brown–Forsythe tests, respectively. Differences between the control and cisplatin-treated groups were analyzed using a two-tailed unpaired Student’s t-test. P < 0.05 was considered statistically significant. Statistical analyses were performed using GraphPad Prism version 9.0 and R version 4.3.2.

## 3 Results

### 3.1 Identification of candidate targets linking cisplatin toxicogenomic responses to AKI

Cisplatin-related toxicogenomic targets were retrieved from the Comparative Toxicogenomics Database, and AKI-associated genes were integrated from OMIM, MalaCards, and the GWAS Catalog. By intersecting these two gene sets, we identified 214 shared genes as candidate targets for cisplatin-induced AKI (Cis-AKI) (Figure 2A). These genes were retained for subsequent network and functional enrichment analyses.

**FIGURE 2:**
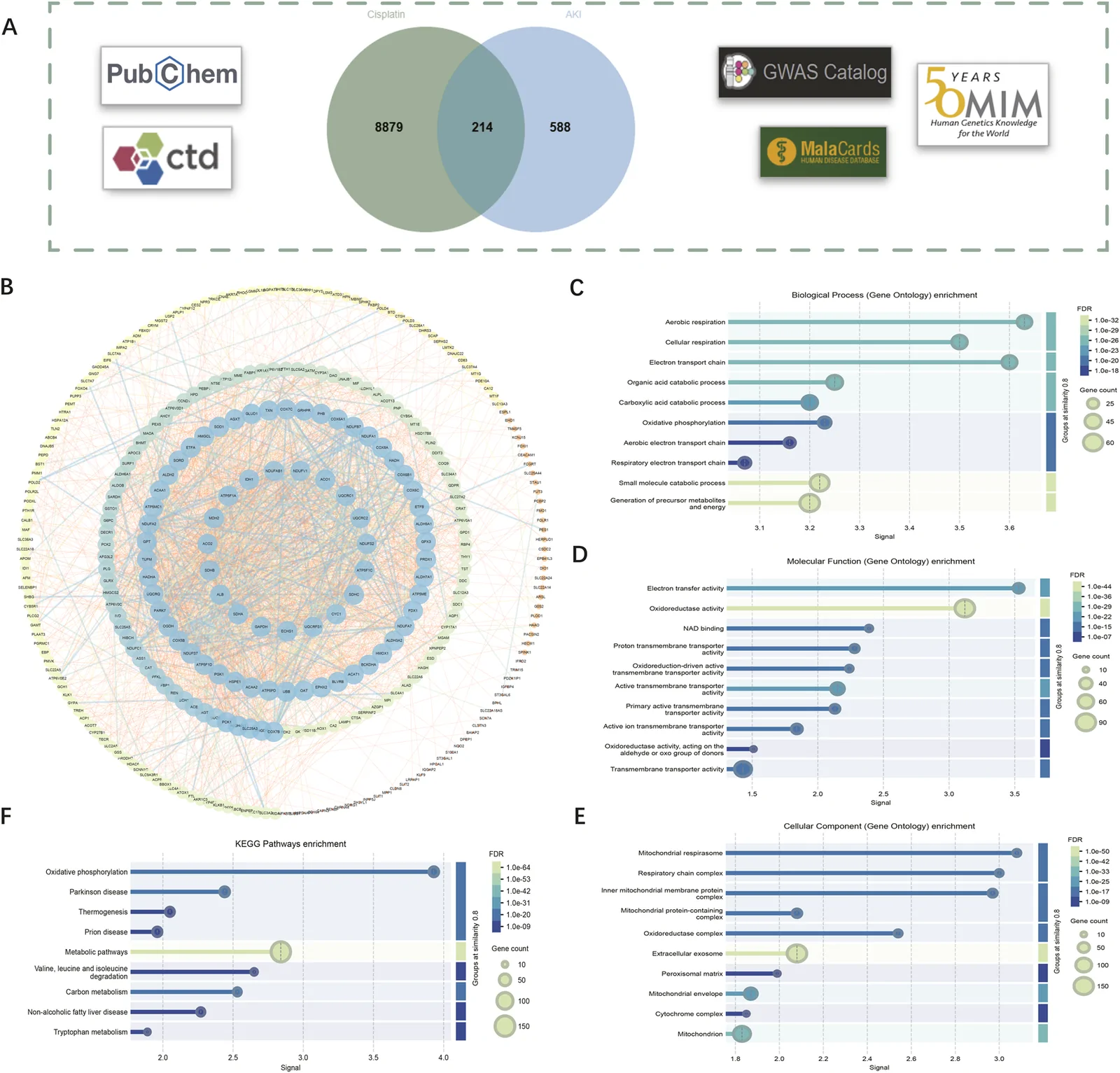
Identification, protein-protein interaction (PPI) network, and functional enrichment analysis of candidate Cis-AKI targets. **(A)** Venn diagram showing the overlap between cisplatin-associated toxicogenomic genes obtained from the Comparative Toxicogenomics Database and AKI-associated genes compiled from OMIM, MalaCards, and the GWAS Catalog. **(B)** Protein-protein interaction network of the 214 candidate targets. **(C–E)** Gene Ontology enrichment analysis for biological process, molecular function, and cellular component categories. **(F)** Kyoto Encyclopedia of Genes and Genomes pathway enrichment analysis. Enriched terms indicate prominent involvement of respiration-related, electron transfer, oxidoreductase, and oxidative phosphorylation pathways.

### 3.2 PPI network and enrichment analyses implicate respiration- and redox-related pathways

To characterize the biological context of the 214 candidate targets, we constructed a protein-protein interaction (PPI) network using STRING and visualized it in Cytoscape (Figure 2B). Functional enrichment analysis showed that the candidate targets were mainly associated with respiration- and redox-related processes. Gene Ontology biological process terms included anaerobic respiration, cellular respiration, and electron transport chain-related processes (Figure 2C). Molecular function terms were enriched for electron transfer activity and oxidoreductase activity (Figure 2D), whereas cellular component terms included mitochondrial respirasome, respiratory-chain complex, and inner mitochondrial membrane protein complex (Figure 2E). KEGG analysis further highlighted oxidative stress- and metabolism-related pathways (Figure 2F). Together, these findings indicate that the candidate targets are mainly involved in mitochondrial respiration, electron transfer, and redox-related processes.

### 3.3 Network analysis prioritizes SDHB and NDUFAB1 as hub genes

To prioritize candidate targets with greater network relevance, we applied eight CytoHubba topological algorithms together with Molecular Complex Detection (MCODE) module analysis (Figures 3A,B). SDHB and NDUFAB1 were consistently ranked among the top candidates across all eight CytoHubba algorithms and were also included in the highest-scoring MCODE module (Figure 3C; Table 1). Based on these results, SDHB and NDUFAB1 were selected as hub genes for subsequent analyses (Table 2). Several additional respiration- or metabolism-related genes, including SDHA, SDHC, NDUFS2, UQCRC1, UQCRC2, ATP5F1A, ATP5F1C, ATP5PD, CYC1, MDH2, GAPDH, and IDH1, were retained as network-prioritized genes. These results indicate that the candidate Cis-AKI network is mainly organized around mitochondrial bioenergetic and redox-related processes.

**FIGURE 3:**
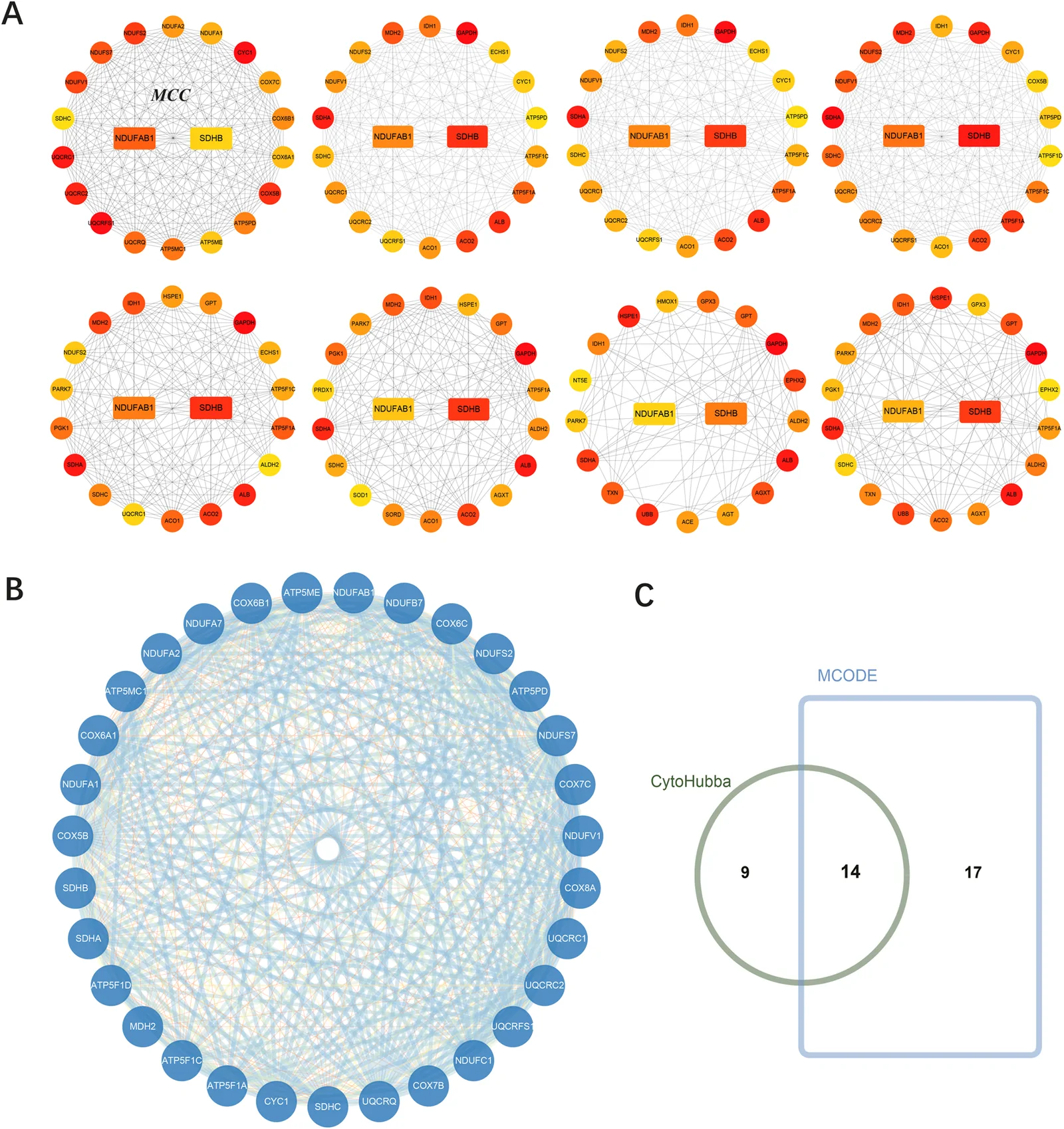
Network-based prioritization of SDHB and NDUFAB1. **(A)** Hub-gene ranking based on eight CytoHubba topological algorithms. **(B)** Highest-scoring module identified by Molecular Complex Detection (MCODE). **(C)** Integrated prioritization of hub genes supported by both CytoHubba ranking and MCODE module membership.

**TABLE 1:** Network-prioritized genes identified by CytoHubba and MCODE module analysis.

Gene	CytoHubba								Score	MCODE
	MCC	MNC	Degree	EPC	Closeness	Radiality	Betweenness	Stress		
SDHB	1	1	1	1	1	1	1	1	8	YES
NDUFAB1	1	1	1	1	1	1	1	1	8	YES
GAPDH	0	1	1	1	1	1	1	1	7	NO
IDH1	0	1	1	1	1	1	1	1	7	NO
SDHA	0	1	1	1	1	1	1	1	7	YES
SDHC	1	1	1	1	1	1	0	1	7	YES
ACO2	0	1	1	1	1	1	0	1	6	NO
ALB	0	1	1	0	1	1	1	1	6	NO
ATP5F1A	0	1	1	1	1	1	0	1	6	YES
MDH2	0	1	1	1	1	1	0	1	6	YES
ACO1	0	1	1	1	1	1	0	0	5	NO
NDUFS2	1	1	1	1	1	0	0	0	5	YES
UQCRC1	1	1	1	1	1	0	0	0	5	YES
ALDH2	0	0	0	0	1	1	1	1	4	NO
ATP5F1C	0	1	1	1	1	0	0	0	4	YES
ATP5PD	1	1	1	1	0	0	0	0	4	YES
CYC1	1	1	1	1	0	0	0	0	4	YES
GPT	0	0	0	0	1	1	1	1	4	NO
HSPE1	0	0	0	0	1	1	1	1	4	NO
NDUFV1	1	1	1	1	0	0	0	0	4	YES
PARK7	0	0	0	0	1	1	1	1	4	NO
UQCRC2	1	1	1	1	0	0	0	0	4	YES

**TABLE 2:** Network characteristics and biological functions of SDHB and NDUFAB1.

Gene	Full name	CytoHubba support	MCODE result	Biological interpretation
SDHB	Succinate dehydrogenase [ubiquinone] iron-sulfur subunit, mitochondrial	8/8 algorithms	Located in highest-scoring module	Complex II/TCA-electron transport interface; candidate node for succinate oxidation, electron transfer, and ROS generation
NDUFAB1	Acyl carrier protein, mitochondrial	8/8 algorithms	Located in highest-scoring module	Mitochondrial acyl-carrier protein associated with respiratory-chain homeostasis and mitochondrial metabolic maintenance

### 3.4 Cisplatin-induced mouse AKI transcriptomic analysis supports respiration- and metabolism-related remodeling

We next analyzed an independent cisplatin-induced mouse AKI transcriptomic dataset to examine the expression patterns of the network-prioritized genes. Because the initial candidate targets were derived from database-based chemical–disease overlap rather than expression-based discovery, this analysis was used to evaluate whether network-prioritized genes showed injury-associated transcriptional remodeling. Under the stringent criterion of FDR <0.05 and log2FC > 1, four candidate hub or supportive genes were significantly upregulated in cisplatin-induced AKI kidneys: Aco2, Aco1, Sdhb, and Aldh2 (Figure 4A). When a relaxed effect-size threshold was applied (FDR <0.05 and |log2FC| > 0.5), fourteen network-prioritized genes were highlighted: Aco2, Sdhc, Aco1, Uqcrc1, Sdhb, Sdha, Atp5f1c, Aldh2, Ndufv1, Uqcrc2, Atp5f1a, Park7, Ndufs2, and Ndufab1 (Figure 4B). These genes were involved in the tricarboxylic acid cycle, respiratory-chain complexes I–V, redox regulation, and mitochondrial metabolism. Notably, Sdhb met the stringent threshold, whereas Ndufab1 was among the genes meeting the secondary threshold. Together, these results showed that cisplatin-induced AKI was accompanied by broad transcriptional changes in respiration-, redox-, and metabolism-related genes.

**FIGURE 4:**
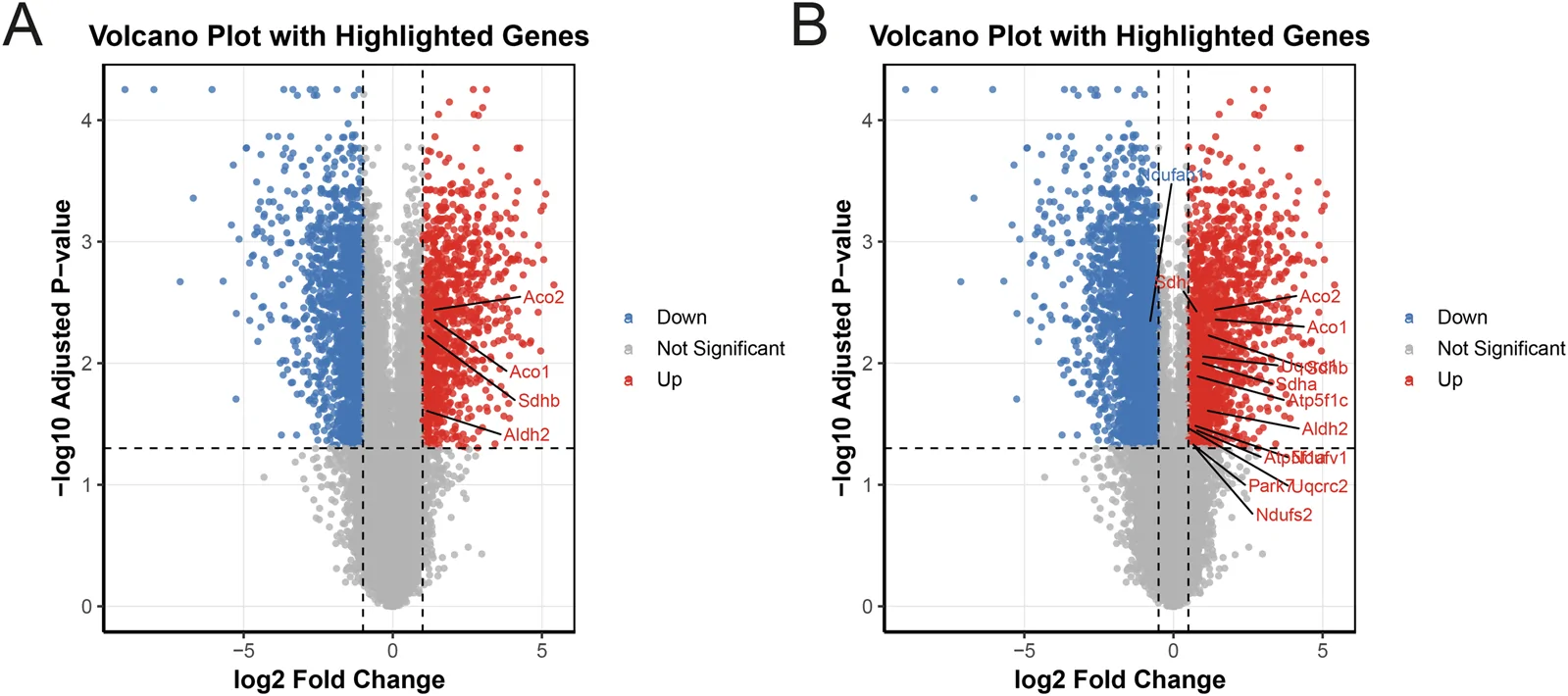
Transcriptomic analysis of network-prioritized genes in cisplatin-induced mouse AKI. **(A)** Volcano plot highlighting four significantly upregulated candidate hub or supportive genes under the stringent threshold of FDR <0.05 and log2FC > 1: Aco2, Aco1, Sdhb, and Aldh2. **(B)** Volcano plot highlighting fourteen network-prioritized genes under the relaxed threshold of FDR <0.05 and |log2FC| > 0.5: Aco2, Sdhc, Aco1, Uqcrc1, Sdhb, Sdha, Atp5f1c, Aldh2, Ndufv1, Uqcrc2, Atp5f1a, Park7, Ndufs2, and Ndufab1.

### 3.5 Single-nucleus transcriptomics reveals cell-type-specific remodeling of SDHB and NDUFAB1

To determine the cellular localization of the two hub genes, we analyzed human kidney single-nucleus transcriptomic data from AKI and normal tissues. After quality control, 20,538 high-quality nuclei were retained and annotated into ten renal cell populations, including immune cells, endothelial cells, podocytes, proximal tubular cells, collecting duct-principal cells, loop of Henle cells, renal progenitor cells, distal convoluted tubular cells, collecting duct-intercalated cells, and stromal cells (Figure 5A). The cellular composition differed between AKI and normal tissues, indicating injury-associated remodeling of the renal cellular landscape (Figure 5B).

**FIGURE 5:**
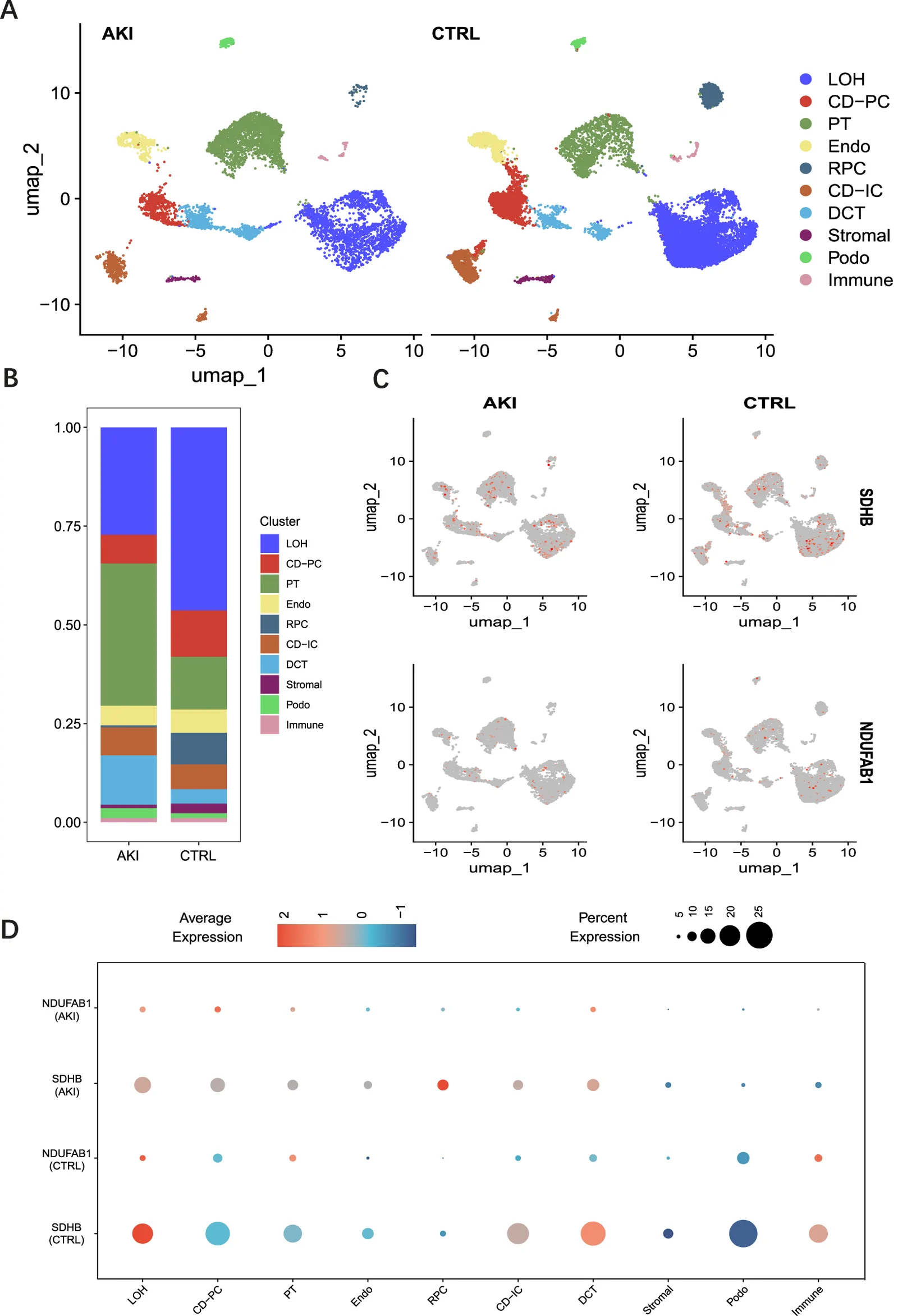
Cell-type-specific expression patterns of SDHB and NDUFAB1 in AKI kidney tissue. **(A)** UMAP visualization of ten annotated renal cell populations, including immune cells, endothelial cells (Endo), podocytes (Podo), proximal tubular cells (PT), collecting duct-principal cells (CD-PC), loop of Henle cells (LOH), renal progenitor cells (RPC), distal convoluted tubular cells (DCT), collecting duct-intercalated cells (CD-IC), and stromal cells. **(B)** Cellular composition of AKI and normal kidney tissues. **(C)** UMAP feature plots showing the distribution of SDHB and NDUFAB1 expression. **(D)** Bubble plot showing average expression and the percentage of expressing nuclei across renal cell types.

SDHB and NDUFAB1 showed distinct cell-type-specific expression patterns across renal compartments (Figures 5C,D). Compared with normal tissues, NDUFAB1 expression was increased in collecting duct-principal cells and distal convoluted tubular cells but decreased in immune cells, proximal tubular cells, and loop of Henle cells. In contrast, SDHB expression was increased in collecting duct-principal cells, proximal tubular cells, endothelial cells, renal progenitor cells, podocytes, and several other cell populations, whereas it was decreased in loop of Henle cells, distal convoluted tubular cells, and immune cell clusters. These bidirectional and compartment-specific changes indicate distinct cell-type-dependent expression patterns of SDHB and NDUFAB1 during AKI, with proximal tubular cells showing a pattern relevant to subsequent trajectory analysis.

### 3.6 Proximal tubular pseudotime analysis indicates AKI-enriched late-state transition

Given the susceptibility of proximal tubular cells to kidney injury, we extracted proximal tubular nuclei for Slingshot-based trajectory analysis. The reconstructed trajectory revealed a continuous transition from early to late pseudotime states (Figure 6A). Control proximal tubular cells were mainly distributed in early trajectory regions, whereas AKI proximal tubular cells were enriched toward late pseudotime states (Figures 6B,C). This distribution suggests that AKI is associated with a continuum of proximal tubular cell states rather than a simple binary separation between normal and injured cells.

**FIGURE 6:**
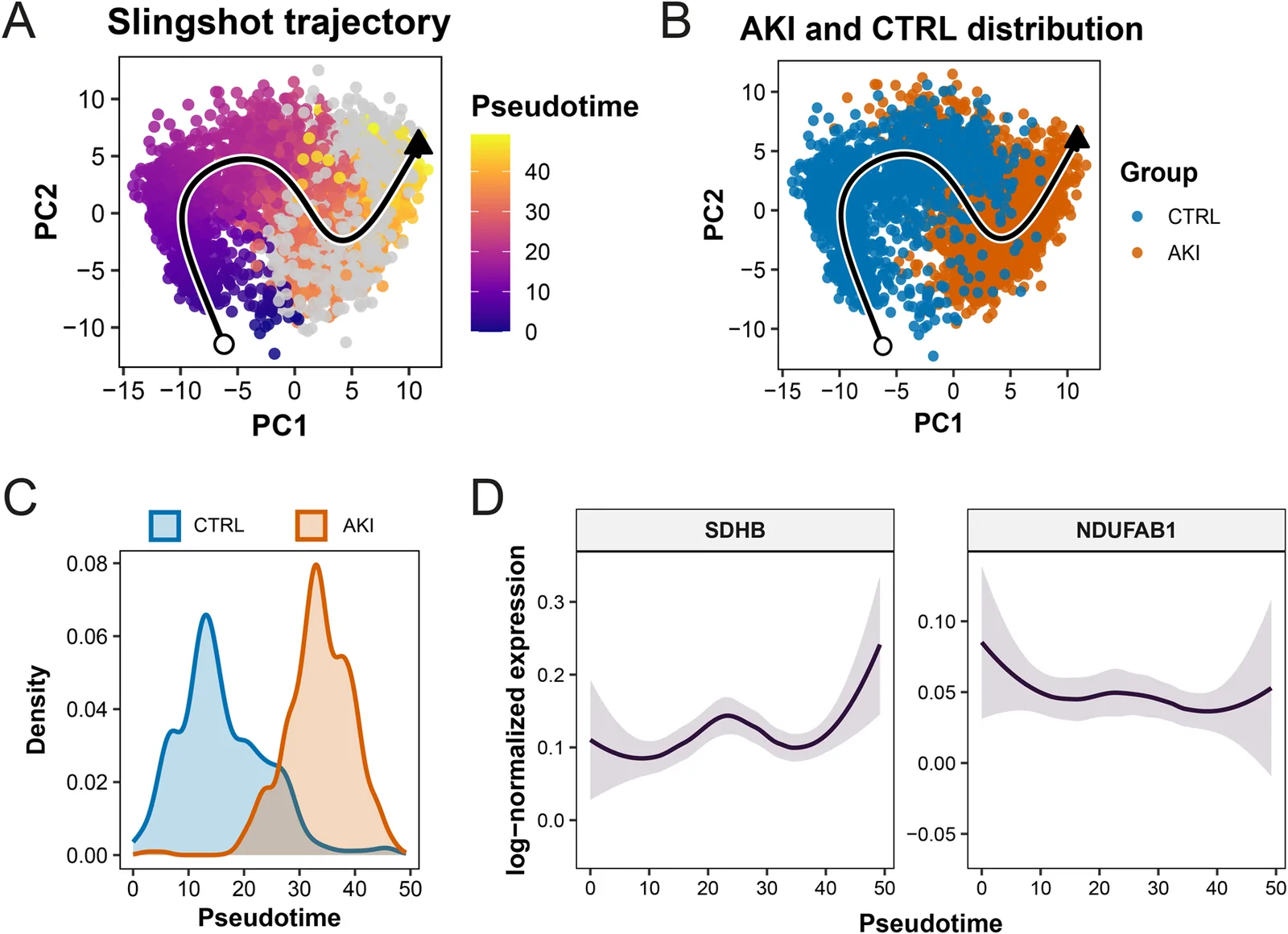
Proximal tubular pseudotime analysis using Slingshot. **(A)** Slingshot-inferred trajectory of proximal tubular cells colored by pseudotime. **(B)** Distribution of AKI and control proximal tubular cells along the trajectory. **(C)** Density plot comparing pseudotime distributions between AKI and control proximal tubular cells. **(D)** Smoothed expression trends of SDHB and NDUFAB1 along pseudotime.

Along pseudotime, SDHB expression gradually increased toward the late trajectory stage, particularly near the terminal portion of the inferred lineage (Figure 6D). By contrast, NDUFAB1 showed a more modest dynamic pattern, with a shallow decline across early-to-middle pseudotime followed by a slight rebound at later states. These results suggest that SDHB is more closely associated with late proximal tubular injury-related states, whereas NDUFAB1 shows weaker and more variable changes along the inferred trajectory.

### 3.7 Targeted *in vitro* expression validation in cisplatin-treated BUMPT cells

To further examine the expression of selected candidate genes in a tubular injury model, qRT-PCR analysis was performed in cisplatin-treated BUMPT proximal tubular epithelial cells. Cisplatin treatment significantly increased the mRNA expression of the tubular injury markers Havcr1/Kim-1 (P = 8.37 × 10^−8^) and Lcn2/Ngal (P = 2.52 × 10^−8^) compared with control cells (Figure 7A). Bcl2 expression was decreased (P = 7.97 × 10^−5^), whereas Casp3 expression was increased (P = 1.22 × 10^−5^) after cisplatin treatment (Figure 7B). Among the six selected candidate genes, Sdhb (P = 3.80 × 10^−6^), Aco2 (P = 1.87 × 10^−5^), Aldh2 (P = 1.85 × 10^−5^), Ndufs2 (P = 1.03 × 10^−4^), and Uqcrc1 (P = 5.36 × 10^−5^) were increased after cisplatin treatment, whereas Ndufab1 was modestly decreased (P = 0.00409) (Figure 7C; Supplementary Table S4). These results indicate that cisplatin-induced tubular injury is accompanied by altered expression of several respiration- and metabolism-related candidate genes in BUMPT cells.

**FIGURE 7:**
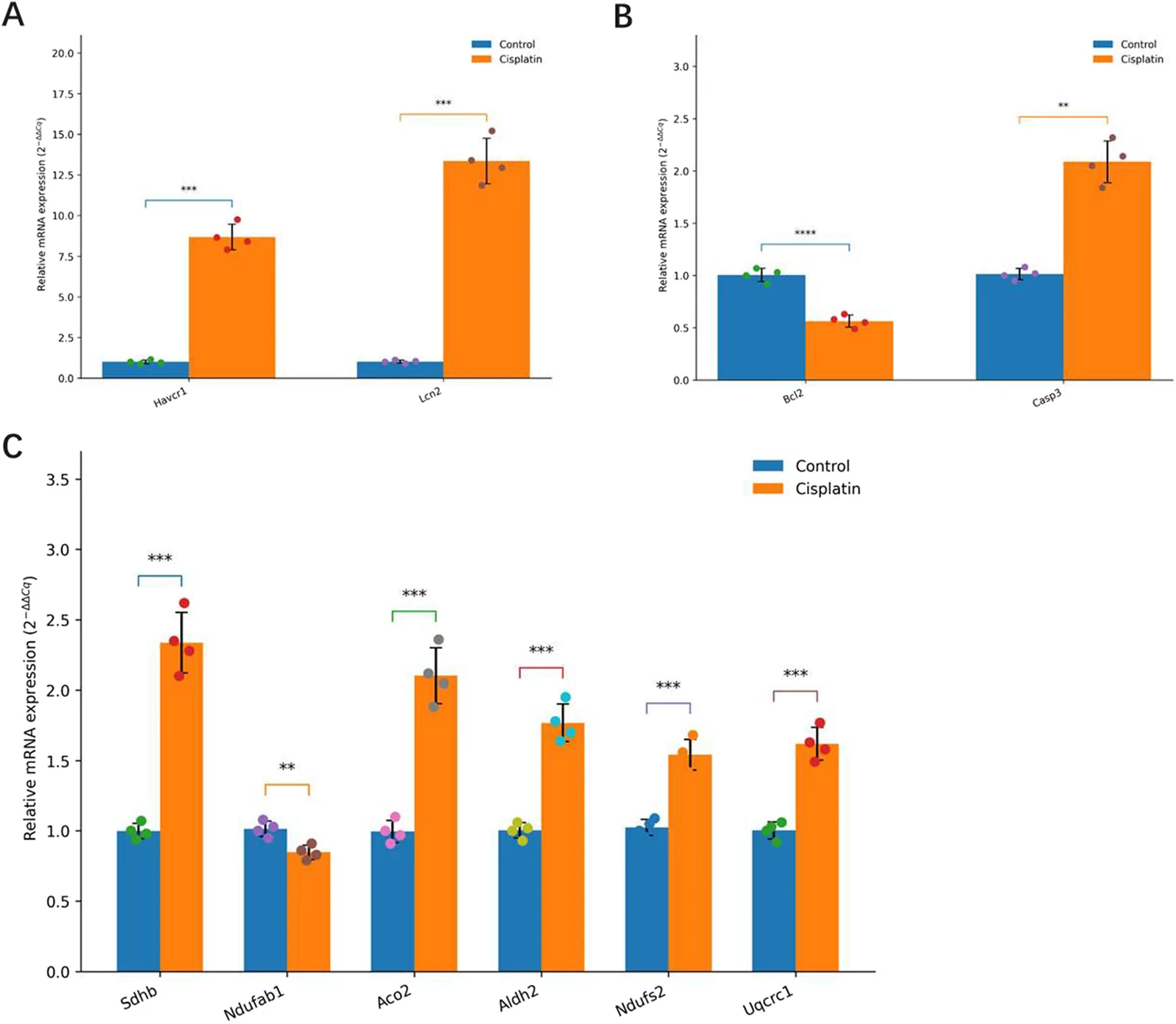
qRT-PCR validation of selected genes in cisplatin-treated BUMPT cells. BUMPT proximal tubular epithelial cells were treated with cisplatin, and relative mRNA expression was measured by qRT-PCR. Four independent biological replicates were included in each group. **(A)** Relative mRNA expression levels of tubular injury markers Havcr1/Kim-1 and Lcn2/Ngal. **(B)** Relative mRNA expression levels of apoptosis-related genes Bcl2 and Casp3. **(C)** Relative mRNA expression levels of selected candidate genes, including Sdhb, Ndufab1, Aco2, Aldh2, Ndufs2, and Uqcrc1. Data are presented as mean ± SD. Statistical significance was determined using a two-tailed unpaired Student’s t-test. *P < 0.05, **P < 0.01, ***P < 0.001.

### 3.8 Mouse knockout phenotype evidence provides genetic context for prioritized targets

To provide additional genetic context for the network-prioritized targets, we reviewed corresponding mouse knockout phenotype annotations in the Mouse Genome Informatics database (Table 3). Phenotype annotations showed that several prioritized targets were linked to renal, metabolic, developmental, or survival-related phenotypes. Notably, Sdhb deficiency was associated with abnormal kidney morphology, whereas Aldh2 deficiency was linked to abnormal urination and systemic metabolic phenotypes. Other respiration- or metabolism-related targets, including Park7, Sdha, Sdhc, Ndufs2, Ndufv1, Uqcrc1, Uqcrc2, Cyc1, and Atp5f1a, were mainly associated with abnormalities in cellular respiration, the tricarboxylic acid cycle, metabolism, development, or survival. Overall, the MGI annotations provided additional biological context for the network-prioritized genes.

**TABLE 3:** Mouse knockout phenotype evidence for network-prioritized targets.

Gene	Model	Impact on specific systems	Phenotypes	MGI ID
Sdhb	Targeted (null/knockout, reporter)	Renal/urinary system, homeostasis/metabolism, embryo/development, mortality/aging, nervous system/vision/behavior, hematopoietic/immune system	Abnormal kidney morphology, decreased mean corpuscular hemoglobin concentration, increased fasting circulating glucose level, preweaning lethality, complete penetrance, decreased neutrophil cell number	MGI:5548548
Ndufab1	Endonuclease-mediated (null/knockout)	N/A	N/A	MGI:7794860
Gapdh	Endonuclease-mediated (null/knockout)	Embryo/development, mortality/aging, nervous system/vision/behavior, hematopoietic/immune system	Embryonic growth retardation, preweaning lethality, complete penetrance, embryonic lethality prior to organogenesis, cataract, abnormal retina morphology	MGI:6423823
Idh1	Targeted (conditional ready, null/knockout)	Homeostasis/metabolism, hematopoietic/immune system, skeleton	Decreased hematocrit, anemia, decreased hemoglobin content, extramedullary hematopoiesis, decreased bone marrow cell number	MGI:5438251
Sdha	Targeted (reporter, null/knockout)	Homeostasis/metabolism, embryo/development, mortality/aging, nervous system/vision/behavior	Increased circulating free fatty acids level, decreased circulating glucose level, increased circulating sodium level, preweaning lethality, complete penetrance, embryonic lethality prior to organogenesis	MGI:5498775
Sdhc	Targeted (reporter, null/knockout)	Embryo/development, mortality/aging	Preweaning lethality, complete penetrance, embryonic lethality prior to tooth bud stage	MGI:5694171
Aco2	Endonuclease-mediated (null/knockout)	Embryo/development, mortality/aging	Preweaning lethality, complete penetrance	MGI:6402258
Alb	Endonuclease-mediated (null/knockout)	Homeostasis/metabolism	Decreased circulating serum albumin level	MGI:5494601
Atp5f1a	Targeted (conditional ready, null/knockout, reporter)	N/A	Decreased body weight, decreased lean body mass	MGI:4432141
Mdh2	Endonuclease-mediated (null/knockout)	Embryo/development, mortality/aging, nervous system/vision/behavior, cardiovascular system	Abnormal embryo size, abnormal embryo turning, embryonic growth retardation, preweaning lethality, complete penetrance, embryonic lethality prior to tooth bud stage	MGI:6257531
Aco1	Targeted (null/knockout)	Homeostasis/metabolism	Abnormal iron homeostasis	MGI:3029223
Ndufs2	Endonuclease-mediated (null/knockout)	Embryo/development, mortality/aging	Abnormal embryo size, preweaning lethality, complete penetrance, embryonic lethality prior to organogenesis, embryonic lethality prior to tooth bud stage	MGI:5754607
Uqcrc1	Targeted (reporter, null/knockout)	Embryo/development, mortality/aging, nervous system/vision/behavior, cardiovascular system, reproductive system	Embryonic lethality, complete penetrance, increased susceptibility to ischemic brain injury, abnormal response to novel object, abnormal behavior, growth/size/body region phenotype	MGI:6403206
Aldh2	Targeted (null/knockout, conditional ready, reporter)	Renal/urinary system, homeostasis/metabolism, skeleton, reproductive system	Abnormal urination, increased circulating free fatty acids level, decreased bone mineral density, decreased circulating LDL cholesterol level, decreased circulating HDL cholesterol level	MGI:4431566
Atp5f1c	Endonuclease-mediated (null/knockout)	N/A	N/A	MGI:8310672
Atp5pd	Endonuclease-mediated (null/knockout)	N/A	N/A	MGI:6314733
Cyc1	Endonuclease-mediated (null/knockout)	Embryo/development, mortality/aging	Decreased embryo size, abnormal embryonic epiblast morphology, abnormal embryonic-extraembryonic boundary morphology, embryonic growth retardation, embryonic lethality between implantation and placentation, complete penetrance	MGI:7425624
Gpt	Targeted (null/knockout, reporter)	N/A	N/A	MGI:3812716
Ndufv1	Targeted (null/knockout, reporter)	Embryo/development, mortality/aging, hematopoietic/immune system	Decreased hematocrit, decreased hemoglobin content, decreased erythrocyte cell number, preweaning lethality, complete penetrance, embryonic lethality prior to organogenesis	MGI:5705868
Park7	Targeted (null/knockout)	Nervous system/vision/behavior, cellular/mitochondrial metabolism	Abnormal cellular respiration, abnormal tricarboxylic acid cycle, nervous system phenotype	MGI:3514165
Uqcrc2	Endonuclease-mediated (null/knockout)	N/A	N/A	MGI:8203407

### 3.9 NicheNet analysis suggests candidate upstream signaling associated with proximal tubular remodeling

To investigate potential intercellular signals associated with AKI-related proximal tubular state transition, we performed NicheNet analysis using proximal tubular cells as receiver cells and AKI-upregulated proximal tubular genes as the gene set of interest. Ligand activity analysis prioritized several candidate upstream ligands potentially regulating AKI-PT transcriptional responses, including TSPAN3, ACE2, PATJ, TGFB1, GPC6, C5, FGF7, EGF, NAMPT, ALCAM, FBN1, COL1A2, ITGA9, and ADAM17 (Figure 8A). Among them, TSPAN3, ACE2, PATJ, TGFB1, and GPC6 showed relatively high ligand activity.

**FIGURE 8:**
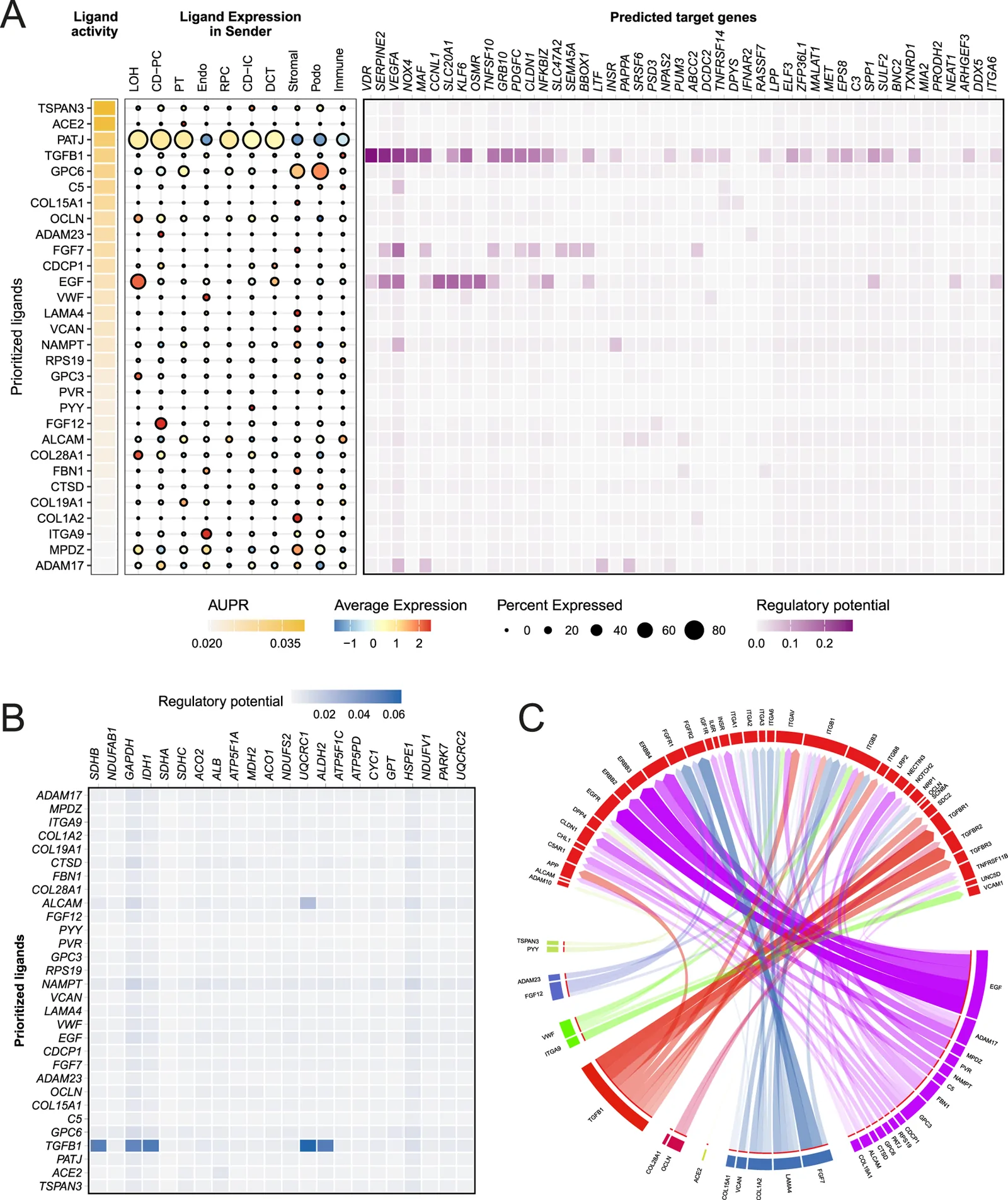
NicheNet analysis of upstream ligand–receptor–target regulatory axes in PT cells. **(A)** NicheNet ligand activity analysis prioritized candidate upstream ligands potentially regulating AKI-induced transcriptional changes in proximal tubular cells. The left panel shows ligand activity ranked by corrected area under the precision-recall curve (corrected AUPR), the middle panel shows ligand expression across sender cell populations, and the right panel shows predicted ligand–target regulatory potential. **(B)** Heatmap showing the predicted regulatory potential of prioritized ligands toward selected network-prioritized respiration- or metabolism-related genes, including SDHB, NDUFAB1, GAPDH, IDH1, UQCRC1, ALDH2, NDUFS2, and related genes. **(C)** Circos plot showing predicted ligand–receptor interactions between prioritized ligands and receptors expressed in proximal tubular cells. Representative axes include TGFB1–TGFBR1/TGFBR2/TGFBR3, EGF–EGFR/ERBB family receptors, FGF7–FGFR family receptors, and C5–C5AR1.

Expression profiling across sender cell populations showed that these prioritized ligands were distributed across multiple renal compartments, including loop of Henle cells, collecting duct cells, proximal tubular cells, endothelial cells, renal progenitor cells, distal convoluted tubular cells, stromal cells, podocytes, and immune cells (Figure 8A). This pattern suggests that AKI-PT transcriptional remodeling may be influenced by multiple renal cell-derived signals rather than a single sender population.

Ligand–target prediction further indicated that TGFB1 and EGF had relatively broad regulatory potential toward AKI-PT target genes, including VDR, SERPINE2, VEGFA, NOX4, KLF6, TNFSF10, PDGFC, CLDN1, NFKBIZ, INSR, SPP1, TXNRD1, MET, and C3. These genes are associated with oxidative stress, inflammatory activation, epithelial injury, repair responses, and metabolic regulation. To connect intercellular signaling with the respiration- and metabolism-related network identified above, we further examined predicted regulatory links between prioritized ligands and selected network-prioritized genes. TGFB1 showed detectable regulatory potential toward SDHB, GAPDH, IDH1, UQCRC1, and ALDH2, whereas ALCAM and NAMPT showed weaker but visible links (Figure 8B).

Ligand–receptor analysis further linked prioritized ligands to receptors expressed in proximal tubular cells, and these inferred interactions were visualized using a circos plot (Figure 8C). Representative inferred axes included TGFB1–TGFBR1/TGFBR2/TGFBR3, EGF–EGFR/ERBB family receptors, FGF7–FGFR family receptors, and C5–C5AR1. Together, these results suggest that upstream ligand–receptor signaling, particularly TGFB1- and EGF-related communication, may contribute to AKI-PT injury responses and respiration-/metabolism-associated transcriptional remodeling.

### 3.10 miRNA-target regulatory network suggests potential post-transcriptional regulation

Using miRDB, we predicted upstream miRNAs targeting SDHB, NDUFAB1, and selected network-prioritized respiration- or metabolism-related genes. Eight miRNAs were predicted to target NDUFAB1, whereas 44 miRNAs were predicted to target SDHB. Several miRNAs showed potential multi-target regulatory relationships. For example, hsa-miR-7-2-3p was predicted to target IDH1, SDHA, and SDHC; hsa-miR-378j was predicted to target GAPDH, SDHA, and SDHC; and hsa-miR-1290 was predicted to target SDHB, SDHA, and SDHC. The miRNA-target network summarized the predicted regulatory relationships among SDHB, NDUFAB1, additional network-prioritized genes, and their candidate upstream miRNAs (Figure 9). These findings suggest that the prioritized Cis-AKI targets may be subject to post-transcriptional regulation, although the predicted miRNA-target relationships require experimental validation.

**FIGURE 9:**
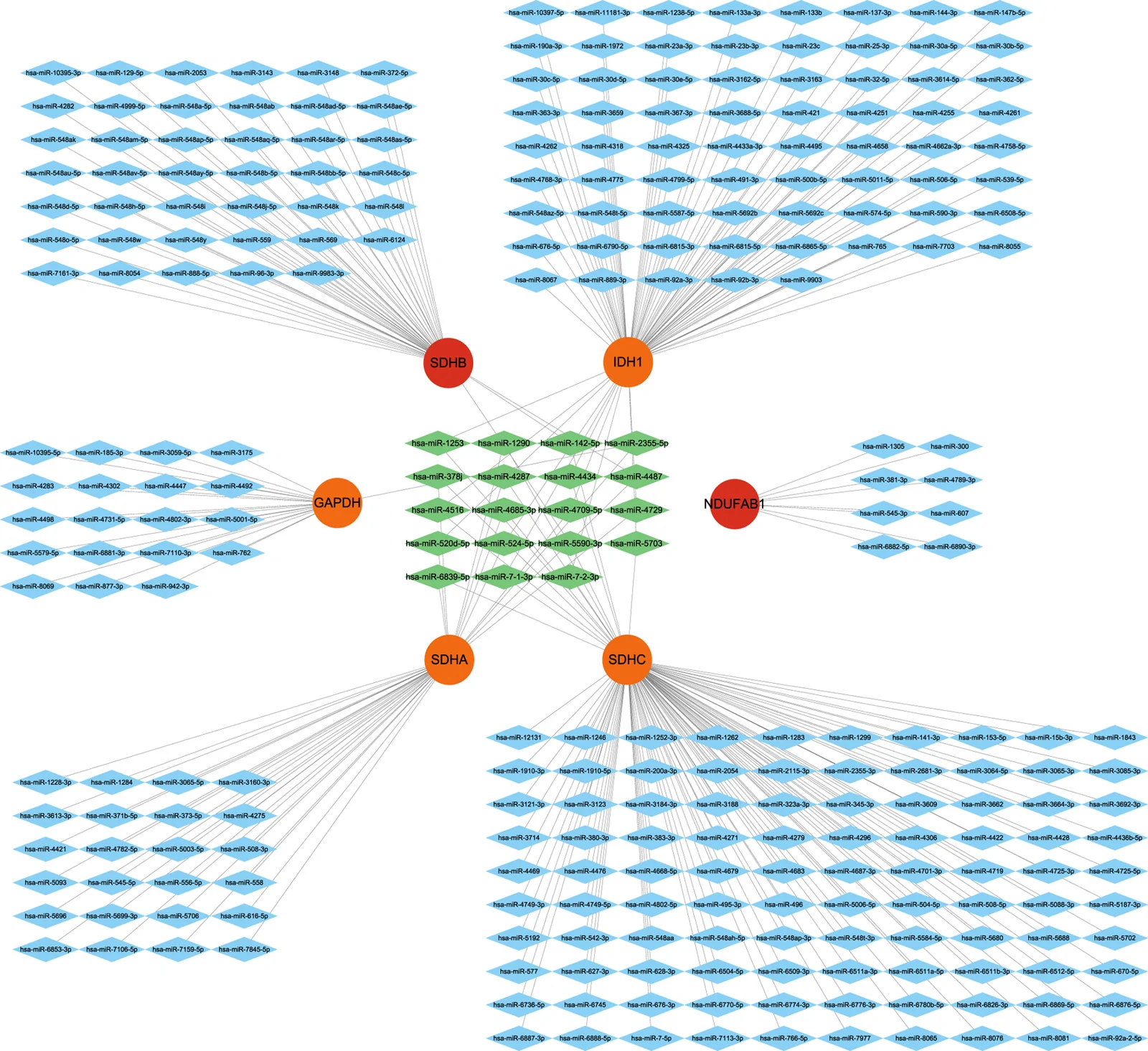
Predicted miRNA-target regulatory network. Red nodes indicate the prioritized hub genes SDHB and NDUFAB1; orange nodes indicate additional network-prioritized respiration- or metabolism-related genes; blue nodes indicate miRNAs predicted to target one gene; green nodes indicate miRNAs predicted to target two or more genes.

### 3.11 Candidate compound annotation and molecular docking

Four compounds annotated in DrugBank as being associated with SDHB or NDUFAB1 were included in the subsequent analysis (Table 4). NDUFAB1-associated compounds included metformin and NADH, whereas SDHB-associated compounds included coenzyme Q1 and 2-Hexyloxy-6-Hydroxymethyl-Tetrahydro-Pyran-3,4,5-Triol (DB04141). Molecular docking was then performed between SDHB or NDUFAB1 and their corresponding candidate compounds. Several docking pairs showed predicted binding energies below −5 kcal/mol, consistent with structural compatibility (Figure 10). These results provide virtual-screening clues for subsequent experimental evaluation of the candidate compounds.

**TABLE 4:** Candidate compounds potentially associated with SDHB and NDUFAB1.

Gene	Full name	Candidate compound	Max phase	Action type	Associated indications/conditions	DrugBank ID
NDUFAB1	Acyl carrier protein, mitochondrial	Metformin	Approved	Inhibitor	Type 2 diabetes; cardiovascular/esrd-related database records	DB00331
​	​	NADH	Approved	Unknown	Parkinson disease; chronic fatigue syndrome; alzheimer disease; cardiovascular disease	DB00157
SDHB	Succinate dehydrogenase [ubiquinone] iron-sulfur subunit, mitochondrial	Coenzyme Q1	Preclinical	Unknown	Not available	DB08689
​	​	2-Hexyloxy-6-hydroxymethyl-tetrahydro-pyran-3,4,5-triol	Preclinical	Unknown	Not available	DB04141

**FIGURE 10:**
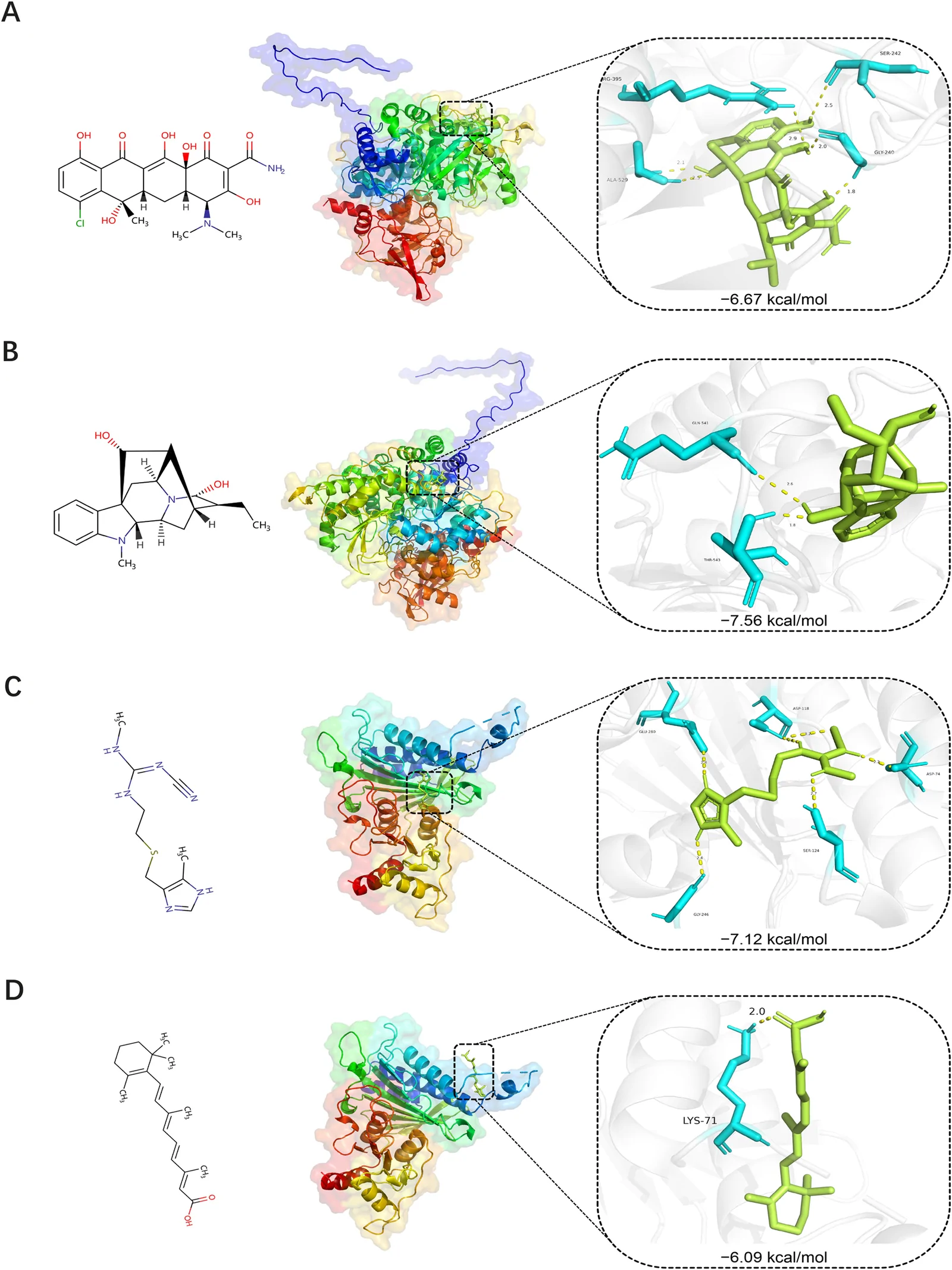
Molecular docking analyses of candidate compounds with SDHB or NDUFAB1. Panels **(A–D)** show four representative docking models, each including the ligand chemical structure, the corresponding protein–ligand complex, and a magnified three-dimensional view of predicted ligand–protein interactions.

Representative lowest-energy docking conformations are shown for SDHB- or NDUFAB1-associated candidate compounds.

## 4 Discussion

This study integrated network toxicology, whole-kidney transcriptomic analysis, human kidney single-nucleus profiling, and targeted *in vitro* expression analysis to investigate respiration-associated molecular changes in cisplatin-induced acute kidney injury (Cis-AKI). The genes shared between cisplatin-associated toxicogenomic records and AKI-related gene sets were mainly enriched in cellular respiration, electron transport, oxidoreductase activity, respiratory-chain complexes, and oxidative phosphorylation. Network analysis further prioritized SDHB and NDUFAB1 within this respiration-associated gene network. Their expression patterns were subsequently examined across whole-kidney, cell-type-resolved, cell-state, and proximal tubular cell models. This integrated approach extends pathway-level observations of mitochondrial dysfunction by prioritizing specific respiration-associated genes and examining their expression across complementary biological levels.

The enrichment of respiration- and redox-related processes is consistent with the biological characteristics of proximal tubular epithelial cells. These cells have high energy requirements and depend heavily on mitochondrial oxidative metabolism to maintain solute transport and epithelial homeostasis. Cisplatin accumulation in proximal tubular cells can induce mitochondrial DNA damage, respiratory-chain dysfunction, ATP depletion, oxidative stress, inflammatory signaling, and regulated cell death. Recent studies further support close coupling between mitochondrial metabolic dysfunction, oxidative stress, and inflammatory injury in AKI (Wu et al., 2024; Wang et al., 2025; Zhou et al., 2025; Zhong et al., 2024; Song et al., 2025; Pang et al., 2026; Ling et al., 2024). For example, loss of mitochondrial pyruvate carrier 2 aggravates cisplatin-induced tubular metabolic dysfunction (Wu et al., 2024), whereas NINJ1-mediated membrane rupture has been linked to amplification of inflammatory responses during AKI (Zhou et al., 2025). Renin–angiotensin signaling has also been implicated in the vascular, hemodynamic, and inflammatory responses to cisplatin nephrotoxicity (Vakilian et al., 2024). Therefore, changes in mitochondrial respiration and redox regulation may represent an important component of the tubular response to cisplatin injury rather than an isolated consequence of cellular damage.

SDHB and NDUFAB1 represent different components of mitochondrial homeostasis. SDHB encodes the iron–sulfur subunit of succinate dehydrogenase and links the tricarboxylic acid cycle to complex II of the electron transport chain (Goetzman et al., 2023). Changes in SDHB expression may influence succinate oxidation, electron transfer, redox balance, and reactive oxygen species production (Shannon et al., 2003; Zeng et al., 2026a). NDUFAB1 encodes a mitochondrial acyl carrier protein involved in iron–sulfur cluster biogenesis and the assembly of respiratory complexes and supercomplexes (Dibley et al., 2020; Hou et al., 2019). The simultaneous prioritization of SDHB and NDUFAB1 therefore highlights two distinct aspects of mitochondrial respiratory homeostasis: complex II-linked metabolism and the organization of respiratory-chain protein complexes (Li G. et al., 2026).

The cisplatin-induced mouse whole-kidney transcriptomic analysis first provided injury-matched support for the respiration-associated gene network. Aco2, Aco1, Sdhb, and Aldh2 met the stringent differential expression threshold, whereas Ndufab1 and several additional respiration- or metabolism-related genes showed more moderate changes. These results indicate that mitochondrial and metabolic genes are broadly remodeled during cisplatin-induced kidney injury, although the magnitude of change differs among individual genes. In particular, Sdhb showed stronger transcriptional changes than Ndufab1, suggesting that the two genes may represent distinct components of the mitochondrial response. Because whole-kidney transcriptomic data reflect multiple renal cell populations, however, these findings alone cannot determine whether the observed changes arise directly from injured proximal tubular cells or from alterations in tissue composition.

Human single-nucleus analysis provided cell-type-specific context for the whole-kidney findings. Although the human dataset represented AKI broadly rather than cisplatin-specific AKI, it showed that SDHB and NDUFAB1 exhibited distinct expression patterns across renal compartments (Gerhardt et al., 2021). In proximal tubular cells, SDHB expression was increased, whereas NDUFAB1 expression was decreased. Increased SDHB may reflect stress-associated remodeling of complex II-linked metabolism or a compensatory transcriptional response. In contrast, reduced NDUFAB1 may be consistent with impaired maintenance of respiratory-complex organization or loss of a homeostatic mitochondrial state. The divergent patterns observed across other tubular and non-tubular populations further indicate that the expression of these genes is strongly dependent on cellular context.

Pseudotime analysis further linked these expression patterns to proximal tubular cell-state changes. AKI proximal tubular cells were enriched toward late pseudotime states, consistent with the gradual transition of tubular cells from relatively preserved to injury-associated states (Higgins et al., 2019). SDHB expression increased toward the later portion of the inferred trajectory, whereas NDUFAB1 showed weaker and more variable changes. These findings suggest that SDHB may be more closely associated with late proximal tubular injury states, while NDUFAB1 may reflect a subtler or stage-dependent component of mitochondrial remodeling. Because pseudotime represents an inferred ordering of cellular states rather than direct chronological progression, these patterns require confirmation in experimental time-course studies.

The qRT-PCR results in cisplatin-treated BUMPT cells further connected the tissue- and single-nucleus-level findings with a proximal tubular epithelial cell model. Cisplatin treatment increased the tubular injury markers Havcr1 and Lcn2, supporting the establishment of tubular injury *in vitro*. Decreased Bcl2 and increased Casp3 expression were also consistent with an apoptosis-associated transcriptional response. Among the selected candidate genes, Sdhb, Aco2, Aldh2, Ndufs2, and Uqcrc1 were increased, whereas Ndufab1 showed a modest decrease. The concordant increase in Sdhb and decrease in Ndufab1 between human proximal tubular cells and cisplatin-treated BUMPT cells strengthens the relevance of these genes to tubular injury. More broadly, the altered expression of genes from the tricarboxylic acid cycle and respiratory-chain complexes suggests that cisplatin induces coordinated mitochondrial transcriptional remodeling in proximal tubular epithelial cells.

The divergent expression patterns of Sdhb and Ndufab1 may reflect their distinct roles within the mitochondrial respiratory system. SDHB is a catalytic component of complex II at the interface between the tricarboxylic acid cycle and electron transport, whereas NDUFAB1 contributes to iron–sulfur cluster biogenesis and the assembly of multiple respiratory complexes and supercomplexes (Goetzman et al., 2023; Hou et al., 2019). Previous studies have shown that loss of NDUFAB1 disrupts respiratory-complex and supercomplex assembly, impairs mitochondrial bioenergetics, and increases ROS production, whereas increased NDUFAB1 expression enhances respiratory capacity and limits ROS generation (Hou et al., 2019). Recent work in proximal tubular AKI has likewise demonstrated that disruption of mitochondrial cristae and respiratory supercomplex organization can accompany impaired mitochondrial respiration (Nicholson et al., 2026). In this context, increased Sdhb expression may represent a stress-associated or compensatory change in complex II-linked metabolism, whereas reduced Ndufab1 may reflect impaired maintenance of respiratory-chain organization. Thus, their opposing transcriptional patterns are compatible with differential remodeling of distinct components of the mitochondrial respiratory machinery during tubular injury. A schematic summary integrating the observed expression patterns of SDHB and NDUFAB1 with their proposed roles in mitochondrial remodeling during proximal tubular injury is shown in Figure 11.

**FIGURE 11:**
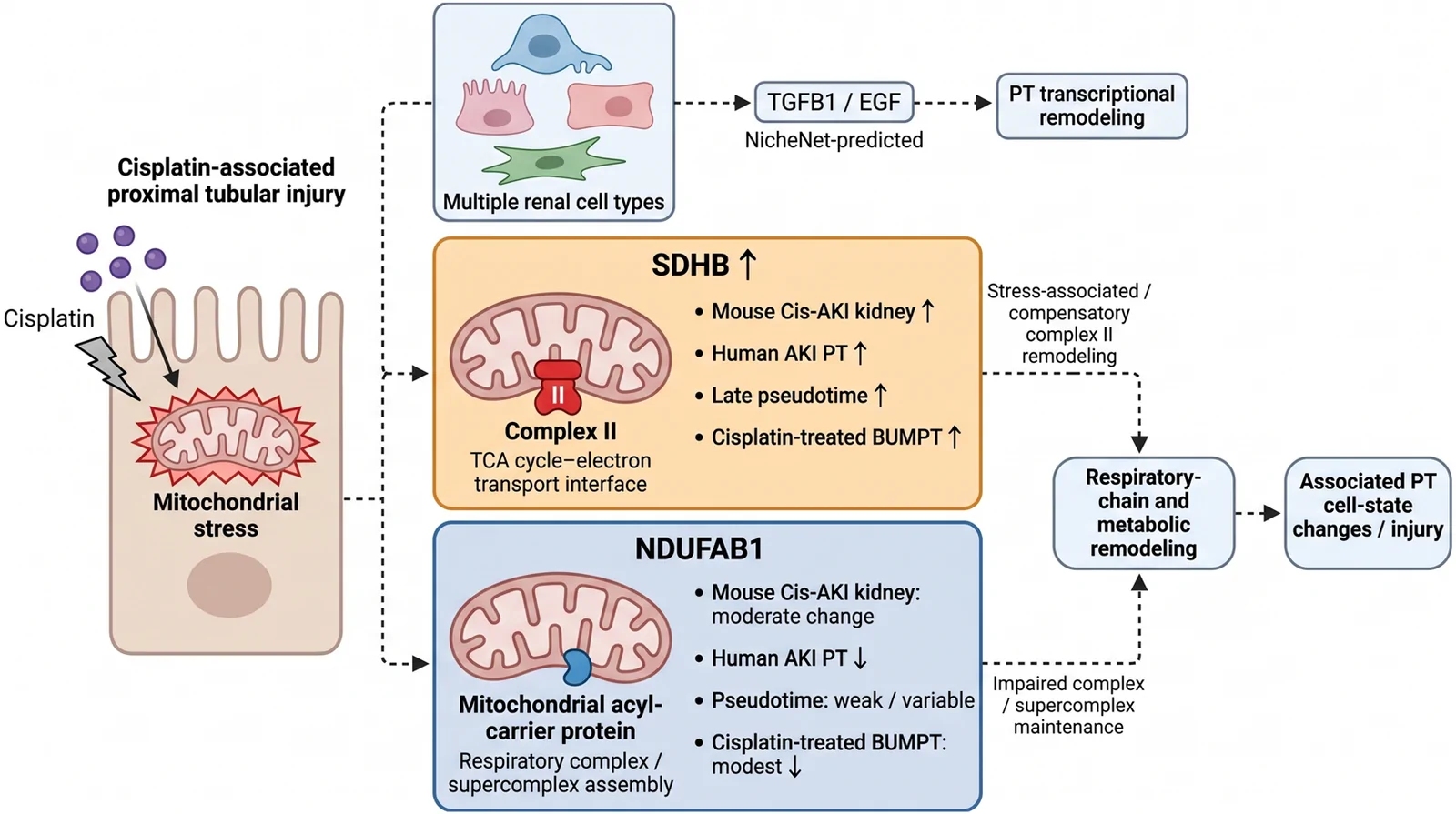
Proposed working model of SDHB- and NDUFAB1-associated mitochondrial remodeling in cisplatin-associated proximal tubular injury. SDHB and NDUFAB1 showed distinct expression patterns across complementary transcriptomic, single-nucleus, pseudotime, and BUMPT evidence. Dashed arrows indicate proposed or computationally inferred relationships.

Mouse knockout phenotype annotations provided additional biological context for the prioritized genes. Sdhb deficiency was associated with abnormal kidney morphology, whereas Aldh2 and several other respiration- or metabolism-related genes were linked to renal, metabolic, developmental, or survival phenotypes (Chen et al., 2023). These findings support the broader importance of the prioritized genes in renal and metabolic biology. However, constitutive knockout phenotypes may reflect developmental abnormalities, systemic metabolic effects, or embryonic lethality and therefore cannot be interpreted as direct evidence of their roles in cisplatin nephrotoxicity.

NicheNet analysis suggested that proximal tubular transcriptional remodeling may also be influenced by upstream intercellular signals (Zhang et al., 2023; Kwon et al., 2023). TGFB1- and EGF-related signaling showed predicted links with AKI-associated proximal tubular gene programs, and TGFB1 was connected to several respiration- or metabolism-related targets. These findings suggest that mitochondrial and metabolic changes in injured proximal tubular cells may be shaped by communication among epithelial, stromal, endothelial, and immune compartments. Nevertheless, these ligand–receptor–target relationships remain computational predictions and require experimental confirmation.

The miRNA and molecular docking analyses provided further candidates for future investigation. Predicted miRNAs may contribute to the post-transcriptional regulation of SDHB, NDUFAB1, and related genes, whereas compound annotation and molecular docking provided exploratory structural clues for selected compounds. These analyses provide candidates for subsequent experimental evaluation but do not establish therapeutic efficacy in Cis-AKI.

The main strength of this study is the progressive integration of whole-kidney, cell-type-resolved, cell-state, and proximal tubular cell evidence. The whole-kidney mouse transcriptomic data identified injury-associated changes in respiration-related genes, the human single-nucleus data localized these changes to specific renal cell populations, pseudotime analysis placed them within proximal tubular state transitions, and the BUMPT experiments examined their expression directly in cisplatin-treated proximal tubular epithelial cells. This design provides a coherent framework for investigating mitochondrial remodeling across complementary biological levels.

Several limitations should be considered when interpreting these findings. First, the toxicogenomic and disease-gene resources used for initial target prioritization integrate heterogeneous evidence and may be influenced by database coverage, annotation quality, and publication bias. Second, the human single-nucleus dataset represented AKI of broader clinical etiologies rather than cisplatin-specific AKI. It therefore provides cell-type and cell-state context for the prioritized genes, whereas the mouse transcriptomic dataset and BUMPT experiments provide the cisplatin-specific validation layers. Cross-species differences may also affect comparisons among these datasets. Third, pseudotime analysis, NicheNet, miRNA prediction, knockout phenotype annotation, and molecular docking are inference-based approaches that require experimental validation. Fourth, the *in vitro* validation was performed in a single proximal tubular cell model with four biological replicates per group and was limited to transcriptional measurements. Although the induction of Havcr1 and Lcn2 and the accompanying changes in Bcl2 and Casp3 supported the establishment of cisplatin-induced tubular injury, the relatively small experimental sample size limits the precision of the expression estimates. In addition, protein abundance or activity of SDHB and NDUFAB1 was not examined, and no gain- or loss-of-function experiments were performed. The present data therefore support the prioritization of SDHB, NDUFAB1, and related respiration-associated genes but do not establish their causal roles in Cis-AKI. Further studies incorporating protein-level validation, targeted gene perturbation, direct mitochondrial functional assays, and *in vivo* models are needed to define their functional roles in cisplatin nephrotoxicity.

## 5 Conclusion

This study prioritized SDHB and NDUFAB1 within a respiration-associated gene network in cisplatin-induced acute kidney injury. Mouse transcriptomic, human single-nucleus, pseudotime, and BUMPT qRT-PCR analyses showed distinct expression patterns of SDHB and NDUFAB1 during kidney and tubular injury. The findings highlight respiratory-chain and metabolic remodeling and proximal tubular cell-state changes in Cis-AKI, while suggesting potential intercellular signaling associated with these responses. SDHB and NDUFAB1 provide candidates for subsequent functional investigation.

## Data Availability

The original contributions presented in the study are included in the article/Supplementary Material, further inquiries can be directed to the corresponding authors.
